# Examining Users’ Adoption of Precision Medicine: The Moderating Role of Medical Technical Knowledge

**DOI:** 10.3390/ijerph17031113

**Published:** 2020-02-10

**Authors:** Xingyuan Wang, Yun Liu, Hongchen Liu

**Affiliations:** School of Management, Shandong University, Jinan 250100, China; liu_yun@mail.sdu.edu.cn (Y.L.); liuhongchen@mail.sdu.edu.cn (H.L.)

**Keywords:** precision medicine, UTAUT model, HBM, privacy risk, adoption intention

## Abstract

Precision medical technologies have received a great deal of attention, but promoting such technologies remains a problem for enterprises and medical institutions. Adopting the unified theory of acceptance and use of technology (UTAUT) model and the health belief model (HBM), this study investigated the key factors affecting users’ willingness to adopt precision medicine (PM) in terms of technical factors and external stimuli. Based on 415 questionnaires, performance expectancy, price value, social influence, and perceived threat of disease were found to significantly increase users willingness to adopt PM; meanwhile, privacy risks had the opposite effect. Knowledge about PM was found to strengthen the positive effect of performance expectancy, price value, social influence, and perceived threat of disease on willingness to adopt PM and weaken the negative effect of privacy risk. This study demonstrates the successful application of UTAUT to the medical field while also providing guidance for the promotion of PM.

## 1. Introduction

Precision medicine (PM) refers to personalized approaches to disease prevention and treatment based on technologies such as genomics, proteomics, and metabolomics [1]. PM application mainly includes molecular diagnosis, gene sequencing, and targeted drug therapy. Previous studies have shown that, compared to other treatment schemes, PM can take into account individual differences, thereby providing a better therapeutic effect [2]. With rapid technological development and people’s increasing attention to health, medical workers and consumers are becoming more interested in PM.

Through a review of the literature on PM, this study found that early research on PM mostly focused on the development and application of new technologies [3,4]. However, the promotion of new PM products and technologies remains a problem for enterprises and medical institutions [5]. Despite the potential benefits and the various PM initiatives in place, recent studies show that the adoption of this service remains insignificant, and many people know little about it. For example, one survey found that although most people in Pennsylvania and Bavaria had heard of PM and genetic testing, they knew little about PM technology and drugs [6]. Since PM developed later in China than in developed countries, such problems are more serious there. First, the combination of technology and clinics is weak; second, patients have an insufficient understanding of PM, and market acceptance is low. The above literature review evidences that PM is promising on its supply side (i.e., providing infrastructure) in China, but little has been done on the demand side (i.e., adoption). It is, therefore, imperative to investigate factors influencing the adoption of PM by citizens. The objective of this study was to attempt to fill the above identified gaps by investigating the adoption and acceptance issues of PM from citizens’ perspectives in a developing country like China and hence, the following specific research question was formulated in this study.

What are the key factors influencing the citizens’ acceptance and adoption of PM in developing countries like China?

At present, most research on the factors that influence the acceptance of emerging technologies is based on the unified theory of acceptance and use of technology (UTAUT). Studies have found, for example, that factors such as performance expectation, effort expectation, social influence, and convenience affect the adoption of mobile medicine [7,8,9], electronic medical services [10], virtual health communities [11], and intelligent medical services [12]. Compared to previous studies, the present one used both the UTAUT model and the health belief model (HBM) to explore the factors affecting consumers’ willingness to use PM. Furthermore, it introduced medical technology knowledge as a moderating variable and examined the effects of the factors under different levels of medical technology knowledge.

## 2. Literature Review

### 2.1. User Acceptance and Adoption of Technology

The technology acceptance model (TAM) was proposed by Davis [13]. TAM suggests that perceived ease of use can not only directly affect consumers’ attitudes but also influence perceived usefulness [13,14]. Perceived usefulness, meanwhile, can not only directly affect willingness to use but also achieve willingness to use by changing users attitudes [13,14]. TAM is often favored in fields such as psychology, management, and sociology because of its high standardization. Some question it, however, because of its oversimplification [15]. Venkatesh and Davis [16], therefore, proposed TAM2, which accounts for factors such as subjective norms, image, and output quality. Those scholars later proposed TAM3, again expanding the factors affecting perceived ease of use and perceived usefulness [17].

UTAUT, meanwhile, provides a very different approach to understanding technology adoption and use. Venkatesh, Morris, Davis, and Davis [18] integrated the independent variables of eight theories, including TAM2 and innovation diffusion theory, to form a model consisting of four core variables (i.e., performance expectancy, social influence, effort expectancy, facilitating conditions) and four moderating variables (i.e., gender, age, experience, voluntary). Venkatesh, Thong, and Xu [19] further proposed UTAUT2, which introduced hedonic motivation, habits, and price values as independent variables. UTAUT and UTAUT2 have been widely used in studies of the adoption and diffusion of information technology. For example, UTAUT has been used to investigate consumers’ use of online shopping platforms [20], electronic banks [21], intelligent robots [22], and social media [23]. Others have applied UTAUT or UTAUT2 models in the medical field to study mobile healthcare [7,8,9], e-health services [10], virtual health communities [11], smart medical services [12], and personal health files [24].

### 2.2. Health Belief Theory

Health belief theory, proposed by Becker [25], is among the most widely used social cognition theories. The earliest version of health belief theory suggested that changes in health behavior might be induced by factors such as perceived susceptibility, severity, benefits, and obstacles, and people would choose their next step based on their knowledge of each factor [26]. Among those factors, perceived susceptibility and perceived severity of disease together constitute the perceived threat of disease [27], which refers to perceptions of how dangerous a disease might be [28,29]. Based on a comparison of health belief theory and social learning theory, Rosenstock et al. [26] argued that self-efficacy should be included as an independent variable in HBM. Health belief theory can predict health-related behaviors and support developing measures to spur healthy behavioral changes [30]. Originally used to detect and prevent unhealthy behaviors, health belief theory is now widely applied to diet modification, health education, disease prevention, and medical technology use. Ahadzadeh, Sharif, Ong, and Khong [31], for example, used HBM and UTAUT models to identify the factors that affect people’s use of health websites, while Tsai [32] used those models to explore what can drive people to embrace telemedicine systems.

## 3. Theoretical Framework and Hypotheses Development

Based on the relevant literature of the UTAUT model, HBM model, and combining with the interview method, this study summarized the factors influencing users to adopt PM. We invited 30 consumers who were aged 34 to 72 to be interviewed for clarifying the influencing factors. We first introduced them to the relevant concepts and application scenarios of PM and then asked them to answer what were the main factors influencing their adoption of PM. The interview is mainly a free interview with no time limit. Based on the answers of 30 consumers, we summarized and sorted the text contents, and combined with relevant literature to finally form five factors influencing the adoption of precision medicine. These five factors fall into two categories. The two categories mainly included technical factors and external stimuli. The five factors mainly included performance expectancy, price value, privacy risk, social influence, and perceived threat of disease. Table 1 shows two categories, five factors and the opinions of some interviewees.

### 3.1. Technical Factors and PM Adoption Intention

Performance expectancy comes from perceived usefulness in TAM and the expected results in the SCT model. Performance expectancy refers to the extent to which individuals believe that using a technology will help them achieve better results [8,18]. PM combines genomics, proteomics, and cutting-edge medical technology to screen biomarkers for people with specific diseases and to classify and treat diseases by detecting such markers [33]. Precision medical treatment is personalized treatment that can potentially prevent or treat cancer and other diseases [34]. Consumer expectation of PM refers to the consumer’s belief that using PM can help prevent or treat diseases and make him or her healthy. Some studies have found that performance expectancy is a key factor in a consumer’s purchase and use of a technology [18,35]. Okazaki, Blas, and Castañeda [36] found that performance expectancy had a positive effect on the willingness to use mobile health. Accordingly, we propose the following hypothesis regarding performance expectancy and PM:

**Hypothesis 1 (H1).**  *Performance expectancy is positively related to users PM adoption intention.*

Similarly, price value, as a dimension of technical characteristics, directly affects consumers’ willingness to use, and it is a convenient and flexible factor in marketing [37]. Previous research on price value has investigated areas such as price psychology, strategies, discounts, frameworks, transparency, and fairness. The present study explored the effect of price rationality on the use of PM. Price value is defined as “the cognitive balance between the perceived benefits and the monetary cost paid by the consumer when purchasing a technology or service” [18]. According to the theory of social exchange, two sides with exchange relations are reciprocal. When one party in an exchange relationship brings benefits to the other, the other party will give corresponding benefits to the former party based on the principle of reciprocity [38]. Therefore, the higher the price value of PM, the more consumers will be willing to accept and adopt it. Thus, H2 is proposed:

**Hypothesis 2 (H2).**  *Price value is positively related to users PM adoption intention.*

Perceived risk refers to uncertainty and expected damage in the outcome perceptions of consumers when purchasing services [39,40]. Privacy risk, as a part of perceived risk, reflects concerns about the disclosure of private information in the purchase of a technology or service [41,42]. In this study, privacy risk refers to consumers’ concerns about personal disclosure in the process of purchasing and using precision medical services. On the one hand, PM based on genomics and proteomics has promoted medical innovation; on the other hand, its databases have attracted attention to matters of privacy [43]. Since October 2009, healthcare organizations have reported 1142 large-scale data breaches to the US Department of Health and Human Services [44]. Moreover, previous studies have found that unclear data policies will generate consumer privacy concerns. For example, consumers may be worried about the unauthorized reuse of their health data by third-party platforms [45]. Many previous studies have found that perceived risk from privacy breaches affects people’s satisfaction and thus affects their willingness to use [46]. Especially for emerging technologies, the risks posed by privacy issues will have a key impact on people’s willingness to use them [47]. Currently, PM, as an emerging technology, has unclear data protection policies; thus, users may perceive a risk of privacy leakage. The higher the perceived privacy risk, the lower the willingness to accept and adopt PM. Thus, the following is proposed:

**Hypothesis 3 (H3).**  *Privacy risk is negatively related to users PM adoption intention.*

### 3.2. External Stimuli and PM Adoption Intention

Social influence comes from the subjective norms in the TRA and TPB models and the social factors in the MPCU model. Social influence refers to a user’s perception of the importance of a person who believes a new technology should be used [18,48]. In this study, social influence refers to the extent to which individuals believe the people around them think they should use PM. Social influences mainly include the opinions of relatives, friends, caregivers, doctors, and other important people [35,48]. Among them, medical personnel, as expert authorities, have more professional and targeted opinions [49], which thereby increase consumers’ understanding of PM. Mazur and Hickam [50] noted that most patients rely on doctors’ advice. Suggestions or information provided by family members and friends as nonprofessionals can improve consumers’ ability to deal with uncertain purchasing decisions [51]. At the same time, consumers always exist in certain social environments and therefore experience pressure from others to follow behavioral norms [52]. Consumers will actively cater to the expectations of surrounding people when making decisions. Thus, the greater the social influence, the more users will actively accept and adopt PM. Therefore, H4 is proposed:

**Hypothesis 4 (H4).**  *Social influence is positively related to users PM adoption intention.*

Perceived threat of disease includes perceived disease susceptibility and perceived disease severity [27]. PM takes into account the variability of individual genes, individual lifestyles, and the environment during treatment [53]. PM can have a significant effect on the prevention or treatment of cancer and cardiovascular diseases. For example, doctors can use early myeloperoxidase to identify early-stage high-risk patients with cardiovascular disease and guide the diagnosis of early coronary artery disease [3]. Doctors can also identify cervical cancer lesions through miR-203 and miR-375 [4]. Survival rates for cancer are generally not high. In the United States, the five-year survival rate for lung cancer is 18%; for pancreatic cancer, it is 8%. Furthermore, the mortality rates of liver, laryngeal, and bladder cancer are increasing [54]. When consumers feel they are susceptible to a disease and believe the disease has serious consequences, they will carefully consider their use options. The more serious the perceived consequences of a disease, the more likely it is that consumers will accept and adopt PM. Therefore, H5 is proposed:

**Hypothesis 5 (H5).**  *Perceived threat of disease is positively related to users PM adoption intention.*

### 3.3. Moderating Effect of Medical Technical Knowledge

User medical technical knowledge refers to relevant knowledge consumers can rely on to solve specific technical problems; it includes both subjective and objective knowledge [55]. User medical technical knowledge influences consumer technical information collection and technical use [56]. When making consumption decisions, consumers tend to judge the attributes of technology based on their own technical knowledge and take this as the basis for making purchasing decisions [57]. Consumers with subjective knowledge make decisions in complex environments based on previous experience [58]. Consumers with objective knowledge tend to actively search for information related to technical attributes and then make decisions [59].

When consumers have high medical technical knowledge, Consumers are more likely to evaluate the efficacy of PM based on its attributes. Performance expectancy also had a higher impact on users PM adoption intention—that is, medical technical knowledge positively regulates the relationship of performance expectancy on PM adoption intention. Therefore, H6a is proposed. Since PM takes into account the genomic characteristics of patients, its cost will rise compared to traditional medicine [60]. Therefore, when consumers know more about PM, their perceived price value will increase, and their willingness to use it will also be higher. that is, medical technical knowledge positively regulates the relationship of price value on PM adoption intention. Therefore, H6b is proposed. On the contrary, when consumers have a better understanding of PM and the protection policies of medical institutions for patients’ privacy, their fear of privacy disclosure will be reduced, which alleviates the negative effect on willingness to use. Therefore, H6c is proposed. When consumers have high medical technical knowledge—that is, they have a better understanding of PM [61]—consumers are more receptive to their advice from relatives, friends and doctors, and social influence has a greater impact on precision medicine technology. Therefore, H6d is proposed. When consumers have higher medical knowledge, they also have a better understanding of the harm of some diseases themselves, that is, the clearer the perceived threat of diseases is, the higher the impact of perceived threat of diseases on the adoption intention of PM will be. Therefore, this paper proposes H6e.

**Hypothesis 6a (H6a).**  *Medical technical knowledge positively regulates the relationship of performance expectancy on PM adoption intention.*

**Hypothesis 6b (H6b).**  *Medical technical knowledge positively regulates the relationship of price value on PM adoption intention.*

**Hypothesis 6c (H6c).**  *Medical technical knowledge negatively regulates the relationship of privacy risks on PM adoption intention.*

**Hypothesis 6d (H6d).**  *Medical technical knowledge positively regulates the relationship of social influence on PM adoption intention.*

**Hypothesis 6e (H6e).**  *Medical technical knowledge positively regulates the relationship of perceived threat of disease on PM adoption intention.*

**Figure 1:**
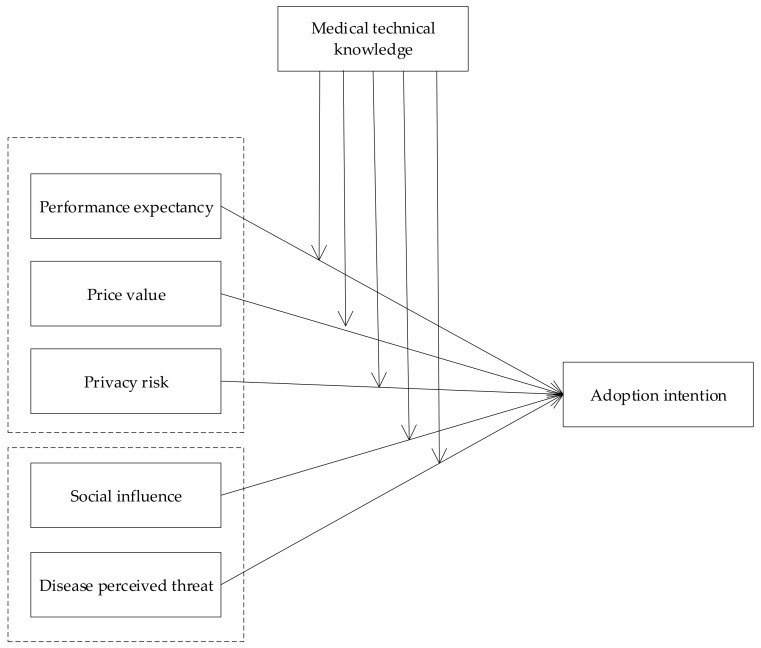
Conceptual model.

Figure 1 shows the conceptual model of this study.

## 4. Research Methodology

### 4.1. Research Design

To test the hypotheses, questionnaires were used to collect data, mainly from middle-aged and older people with a high risk of disease. We hired a professional research institute in China that has a large number of potential respondents. To improve the credibility and validity of the sample, we offered financial incentives to encourage participation. A total of 550 questionnaires were collected.

Questionnaires with the following characteristics were considered invalid: (1) questionnaires with exactly the same answers for 10 consecutive questions and (2) questionnaires with missing values. In the end, 415 valid questionnaires were obtained (recovery rate: 75.45%). The ratio of sample size to measurement items was 17.29:1, which is above the commonly accepted threshold of 10:1 [62]. The statistical information of the samples is as Table 2.

As shown in Table 2, 194 men (46.747%) and 221 women (53.253%) participated in the survey; thus, the proportion of men and women was not too imbalanced. In terms of age distribution, 26 people were below age 30 (6.265%), 41 were 31–40 (9.880%), 54 were 41–50 (13.012%), 164 were 51–60 (39.518%), 121 were 61–70 (29.157%), and 9 were 71 or above (2.169%). People aged 51–70, who are the most likely to get sick, accounted for 68.675% of the total. In terms of occupation, respondents were mainly white-collar workers (61, or 14.699%) and ordinary workers (275, or 66.265%). Finally, respondents’ monthly income was mainly in the range of 0–8000 yuan (77.59%)

### 4.2. Measurement

The questionnaire consisted of 28 questions divided into seven categories: performance expectancy, price value, privacy risk, social influence, perceived threat of disease, users product knowledge, and adoption intention. Basic information about the sample was included at the end of the questionnaire. Performance expectancy included four items based on the scales of Hoque and Sorwar [8] and Venkatesh et al. [18]. Price value included three items based on Venkatesh et al. [18] and Duarte and Pinho [63]. Privacy risks include three questions based on the scales of Milne and Culnan [42] and Ghosh and Swaminatha [64]. Social influence included four items based on Venkatesh et al. [18] and Macedo [35]. Perceived threat of disease included three items drawn from Kahsay, Hiluf, Shamie, Tadesse, and Bazzano [65] and Restivo et al. [66]. User’s medical technical knowledge included three items derived from Hairong Li, Daugherty, and Biocca [67]. Adoption intention included four items based on Cheung et al. [47] and Venkatesh et al. [18]. Seven-point Likert scales were used for all items.

## 5. Data Analysis and Results

### 5.1. Measurement Model

SPSS 25.0 was used to analyze the reliability and validity of the model through confirmatory factor analysis (CFA). Cronbach’s alpha and CR values were used to measure the reliability of the questionnaire; convergent validity and discriminant validity were used to determine validity. Specifically, Cronbach’s alpha coefficients of performance expectancy, price value, privacy risk, social influence, perceived threat of disease, medical technical knowledge, and adoption intention were 0.923, 0.877, 0.900, 0.921, 0.896, 0.900, and 0.909, respectively, which are all higher than 0.7; the CR values were 0.938, 0.920, 0.931, 0.934, 0.915, 0.937, and 0.848, respectively, which are all greater than 0.7. Thus, the scale had good reliability (Table 3)

Since all scales were mature, reliability was reasonably good. The factor loadings of the measurement items of each variable were between 0.630 and 0.930. The average variance extracted (AVE) values for performance expectancy, price value, privacy risk, social influence, perceived threat of disease, medical technical knowledge, and adoption intention were 0.791, 0.793, 0.818, 0.780, 0.782, 0.831, and 0.585, respectively, which are all greater than 0.5, indicating good convergent validity. Meanwhile, the AVE square roots of performance expectancy, price value, privacy risk, social influence, perceived threat of disease, medical technical knowledge, and adoption intention were 0.889, 0.890, 0.904, 0.883, 0.884, 0.912, and 0.765, respectively, which are all higher than their correlation coefficients with other variables, indicating that the model had good discriminant validity. Therefore, the questionnaire has good validity (see Table 3 and Table 4).

### 5.2. Correlation Analysis

Table 4 shows the mean, standard deviation, and correlation coefficients between variables. The correlation coefficients of performance expectancy, price value, privacy risk, social influence, perceived threat of disease, and adoption intention were 0.336, 0.142, −0.216, 0.407, and 0.425, respectively, and all are significant. There were significantly positive correlations between performance expectancy, price value, social influence, perceived threat of disease, and adoption intention, while there were significantly negative correlations between privacy risk and adoption intention.

### 5.3. Results

SPSS 25.0 was used to verify relationships between variables; Table 5 shows the results. The dependent variable in Model 1 and Model 2 is PM adoption intention. Model 1 includes four control variables: age, gender, occupation, and monthly income. Model 2, based on Model 1, introduces five independent variables: performance expectancy, price value, privacy risk, social influence, and perceived threat of disease. In order to exclude collinearity, this study made collinearity diagnosis for model 1-model 2 in this paper, in which the VIF (Variance Inflation Factor) of all coefficients was less than 2, so there was no multicollinearity.

It can be seen from Model 2 in Table 5 that performance expectancy, price value, privacy risk, social influence, and perceived threat of disease was positively related to PM adoption intention. Privacy risks was negatively related to PM adoption intention. Specifically, performance expectancy had a positive correlation with PM adoption intention, and the standardization regression coefficient was 0.397 (*p* < 0.001). Price value had a positive correlation with PM adoption intention, and the standardization regression coefficient was 0.148 (*p* < 0.001). Privacy risks had a negative correlation with PM adoption intention, and the standardization regression coefficient was −0.295 (*p* < 0.001). Social influence had a positive correlation with PM adoption intention, and the standardization regression coefficient was 0.436 (*p* < 0.001). Perceived threat of disease was significantly positively correlated with PM adoption intention, with a standardized regression coefficient of 0.448 (*p* < 0.001). Therefore, H1, H2, H3, H4, and H5 are all supported.

We tested the moderating effect of medical technical knowledge by consulting previous studies. First, the independent variables performance expectancy, price value, privacy risk, social influence, and perceived threat of disease and the moderator variable medical technical knowledge were standardized; then, interactive items were constructed. The purpose of standardization is to reduce the problem of multicollinearity in the regression equation. In this study, Models 3 and 4 were used to verify the moderating effect of product knowledge; Table 5 shows the results. Model 3 introduces user medical technical knowledge on the basis of Model 2. Based on Model 3, Model 4 introduces the interaction terms after the standardization of the independent variables and moderator variables. In order to exclude collinearity, this study made collinearity diagnosis for model 3-model 4 in this paper, in which the VIF (Variance Inflation Factor) of all coefficients was less than 2, so there was no multicollinearity.

As can be seen from Model 4, performance expectancy had a significantly positive correlation with PM adoption intention, with a standardized regression coefficient of 0.427 (*p* < 0.001). The interaction between performance expectancy and medical technical knowledge had a significantly positive correlation with PM adoption intention, with a standardized regression coefficient of 0.088 (*p* < 0.01). Medical technical knowledge enhanced the positive effect of performance expectancy on PM adoption intention; thus, H6a is supported. Price value had a greatly positive correlation with PM adoption intention, with a standardized regression coefficient of 0.161 (*p* < 0.001). The interaction between price value and product knowledge had a significantly positive correlation with PM adoption intention, with a standardized regression coefficient of 0.148 (*p* < 0.001). Medical technical knowledge strengthened the positive effect of price value on PM adoption intention; thus, H6b is supported. Privacy risk had a significantly negative correlation with PM adoption intention, with a standardized regression coefficient of −0.292 (*p* < 0.001). The interaction between privacy risk and medical technical knowledge had a significantly positive correlation with PM adoption intention; the standardized regression coefficient was 0.163 (*p* < 0.01). Medical technical knowledge undermined the negative effect of privacy risks on PM adoption intention; thus, H6c is supported. Social influence had a significantly positive correlation with PM adoption intention, with a standardized regression coefficient of 0.431 (*p* < 0.001). The interaction between social influence and medical technical knowledge had a significantly positive correlation with PM adoption intention, with a standardized regression coefficient of 0.084 (*p* < 0.01). Medical technical knowledge strengthened the positive effect of social influence on PM adoption intention; thus, H6d is supported. Perceived threat of disease had a significantly positive correlation with PM adoption intention, with a standardized regression coefficient of 0.468 (*p* < 0.001). The interaction between perceived threat of disease and medical technical knowledge had a significantly positive correlation with PM adoption intention, with a standardized regression coefficient of 0.263 (*p* < 0.001). Medical technical knowledge strengthened the positive effect of perceived threat of disease on PM adoption intention; thus, H6e is supported.

## 6. General Discussion

### 6.1. Principal Results

The study used a combination of UTAUT and HBM models to explore the key factors affecting PM adoption intention. Performance expectancy and perceived threat of disease were found to be positively related to PM adoption intention. Specifically, the higher the user’s functional perception of PM, and the greater the fear of disease, the higher the PM adoption intention. This is consistent with the findings of UTAUT model and HBM model in the study of other technology [10,31].

Social influence was found to be positively related to PM adoption intention. This suggests that for the middle-aged and elderly, the advice of relatives and friends can encourage them to accept and adopt PM. The results may have something to do with the Chinese being influenced by the doctrine of the mean (Zhong-Yong, which is a form of Confucianism). Previous studies have shown that in interpersonal relationships, individuals with a higher level of Zhong-Yong beliefs tend to analyze the environment and the feelings of others, choose favorable ways for themselves and others [68], and are more likely to be influenced by others.

Privacy risk was negatively related to PM adoption intention. However, the impact of privacy risks on Chinese consumers’ adoption of PM is relatively low. The results may have something to do with Chinese people’s low awareness of privacy. Some studies point out that few people realize that they need to protect their privacy by some means in China [69].

Price value were found to be positively related to PM adoption intention. This is consistent with the results of previous studies. Specifically, the more reasonable the price perception, the higher the PM adoption intention. Reducing the price is a way to increase the price value, and the government’s inclusion of relevant tests of PM into the Chinese Urban Resident Basic Medical Insurance (URBMI) can effectively reduce the price of PM. Previous studies have shown that URBMI is effective in reducing financial barriers to treatment in people of moderate income in China [70].

Medical technical knowledge played a positive moderating role in the effects of expectation, price value, social impact, and perceived threat of disease on PM adoption intention. However, it played a negative moderating role when privacy risk was the independent variable. That is to say, for Chinese middle-aged and elderly users, with their purchasing power, they are more likely to trust their relatives’ opinions. With certain knowledge of PM, it is easier to accept precision medicine technology for them.

### 6.2. Implications

This study’s theoretical significance is mainly reflected in the following: First, HBM was mainly used to examine external environmental factors that affect people’s health behavior changes. The UTAUT model was primarily used to explore the technical characteristics that influence people’s use of information technology and products. This study integrated HBM and UTAUT to explain and predict PM adoption intention in terms of external stimulus and product factors. Compared to previous studies, this research made sure to consider multiple factors. Second, past research has mainly applied the UTAUT model in the field of information technology (e.g., online shopping platform adoption, electronic banking, intelligent robots). While some studies have introduced UTAUT into the medical field, exploring the influencing factors of e-health and mobile medicine, those are still within the scope of information technology. PM is not quite the same as information technology. PM proposes using biotechnology to personalize treatment for people with specific diseases. This study used the UTAUT model to explore the factors affecting PM adoption intention and expand the application of the UTAUT model in the medical field. Third, this study not only considered the effect of technical characteristics and external environment on PM adoption intention but also introduced user medical technical knowledge. It also explored how different factors will affect PM adoption intention under different medical technical knowledge levels, upgrading the UTAUT model.

This study’s management implications are mainly reflected in the following two aspects. First, this study found that performance expectancy, price value, social influence, and perceived threat of disease were positively related to PM adoption intention, while privacy risk had the opposite effect. Therefore, for medical institutions, users PM adoption intention can be improved in the following ways: (1) Medical institutions should improve users functional perceptions of PM so they can fully understand the therapeutic effects of PM; (2) In the early stages of PM development, proper disclosure of R&D funding can improve consumer expectations and thus promote a higher usage intention; therefore, medical institutions should appropriately announce R&D funding for PM; (3) On this basis, PM technology should be constantly improved to reduce costs and thus prices, so that patients can get standardized treatment. Medical institutions can also give play to the positive influence of their important relevant personnel on users through doctor recommendations; (4) Medical institutions can also use anxiety marketing to publicize the harms caused by diseases to promote the purchase and use of PM, and lastly; (5) Medical institutions should take measures to strengthen the protection of patients’ data to prevent the disclosure of private information. Furthermore, this study found that users medical technology knowledge will strengthen the positive relationship of performance expectancy, price value, social influence, and perceived threat of disease on PM use intention and weaken the negative relationship of privacy risk on PM use intention. Therefore, medical institutions should enhance users understanding of diseases and medical technology and actively publicize the harm of diseases and relevant PM knowledge through public service advertisements and community medical services.

### 6.3. Limitations and Further Research

This study has some limitations. First, most respondents were recruited from a platform, and there were only 415 valid samples. Future studies can further investigate patients in hospitals and expand the sample to further verify the generalizability of this study’s findings.

Second, this study only considered users PM adoption intention. Whether PM can be promoted and applied largely depends on doctors’ recommendation and adoption. Future research can explore the key factors affecting doctors’ recognition and adoption of PM and compare the factors affecting users PM adoption intention.

Finally, since PM is more tailored and personal, it requires more patient decision-making than when a doctor prescribes nonprecision treatment. Future research can further explore the effect of social influence on PM and non-PM for comparison.

## 7. Conclusions

Based on consumers’ subjective perceptions of precision medicine (PM), this study used the health belief model (HBM) and UTAUT model and tested the key factors affecting PM adoption intention using SPSS 25.0. Performance expectancy, price value, social influence, and perceived threat of disease were found to be positively related to PM adoption intention. Privacy risk was negatively related to PM adoption intention. Additionally, knowledge about PM was found to strengthen the positive effect of performance expectancy, price value, social influence, and perceived threat of disease on willingness to adopt PM and weaken the negative effect of privacy risk. This study demonstrates the successful application of UTAUT to the medical field while also providing guidance for the promotion of PM.

## Figures and Tables

**Table 1:** Factors influencing the adoption of PM: consumer supporting quotes.

Category	Factor	Consumer Quotes
Technical factors	Performance expectancy	For me, the main question is whether PM has a good effect and whether it can cure the disease. (interviewee 5) It is mainly to see whether PM has better therapeutic effect than traditional medicine. (Interviewee 7) Whether PM has a better therapeutic effect and whether it can better treat diseases is my most concern. (Interviewee 14) …
	Price value	The cost of PM will affect whether I use it or not. (Interviewee 10) I focus on the price of PM. (Interviewee 19) Whether precision medicine is included in Chinese Urban Resident Basic Medical Insurance (URBMI) is the factor that affects whether I accept PM, because the general price of PM is relatively high. (Interviewee 25) …
	Privacy risk	I don’t use PM. I’m afraid my privacy will be compromised. (Interviewee 4) I’m not going to adopt PM. As in the novel, the relationship between genetic information and wealth, status, health, and even life, the adoption of PM may lead to higher insurance costs, borrowing risks, targeted marketing, and genetic discrimination. (Interviewee 6) There are mainly the following points: 1. How to protect the privacy of patients, and will the medical results cause discrimination by insurance companies or employers? … (Interviewee 17) …
External stimuli	Social influence	The recommendations of doctors and relatives are also more critical. My father has advanced lung cancer. Now doctors recommend chemotherapy or genetic testing to take targeted medicines. With my support, my father chose genetic testing to take targeted medicines. (Interviewee 8) I will only consider using PM when I see real cases of successful use of PM around me. (Interviewee 9) When choosing between traditional treatment and PM, I tend to consider my family’s suggestions. (Interviewee 16) …
	Perceived threat of disease	The worry about diseases is the main reason why I choose PM. Because my family has a genetic history, I want to know the probability of the disease in the next generation through the test. (Interviewee 2) For general diseases, I will definitely not choose PM. When I am faced with cancer, I will choose PM. At this time, I will not consider the price. (Interviewee 3) When this disease seriously threatens my life, I will choose PM. (Interviewee 11) …

**Table 2:** Demographic information.

Demographic Variable		Number	Percentage (%)
Gender	Male	194	46.747
	Female	221	53.253
Age	31 or less	26	6.265
	31–40	41	9.880
	41–50	54	13.012
	51–60	164	39.518
	61–70	121	29.157
	71 or more	9	2.169
Occupation	Corporate white collar	61	14.699
	Ordinary worker	275	66.265
	Civil servant	30	7.229
	Teacher	33	7.952
	Other	16	3.855
Monthly income	0–3000 yuan	116	27.951
	3000–5000 yuan	147	35.422
	5000–8000 yuan	59	14.217
	8000–10,000 yuan	69	16.627
	Over 10,000 yuan	24	5.783

**Table 3:** Reliability and validity analysis.

Variable	Measurement Index	Factor Loading	Cronbach α	CR	AVE
Performance expectancy (PE)	PE1	0.874	0.923	0.938	0.791
	PE2	0.902			
	PE3	0.891			
	PE4	0.891			
Price value (PV)	PV1	0.880	0.877	0.920	0.793
	PV2	0.907			
	PV3	0.885			
Privacy risk (PR)	PR1	0.891	0.900	0.931	0.818
	PR2	0.930			
	PR3	0.891			
Social influence (SI)	SI1	0.879	0.921	0.934	0.780
	SI2	0.877			
	SI3	0.896			
	SI4	0.880			
Perceived threat (PT)	PT1	0.884	0.896	0.915	0.782
	PT2	0.905			
	PT3	0.864			
Medical technical knowledge (MTK)	MTK1	0.908	0.900	0.937	0.831
	MTK2	0.929			
	MTK3	0.898			
Adoption intention (AI)	AI1	0.741	0.909	0.848	0.585
	AI2	0.630			
	AI3	0.789			
	AI4	0.878			

**Table 4:** Correlation analysis.

Title	Mean	SD	PE	PV	PR	SI	PT	MTK	AI
PE	4.545	1.654	**1**						
PV	4.678	1.611	–0.078	**1**					
PR	4.551	1.700	0.009	0.007	**1**				
SI	4.682	1.605	–0.034	–0.051	0.085	**1**			
PT	4.591	1.684	–0.079	0.107 *	0.077	0.039	**1**		
MTK	4.347	1.759	–0.023	0.055	0.004	–0.017	–0.058	**1**	
AI	4.658	1.527	0.336 **	0.142 **	–0.216 **	0.407 **	0.425 **	–0.036	**1**

** significant at the 0.01 level, * significant at the 0.05 level.

**Table 5:** Hypothesis testing.

Variable Types		Model 1	Model 2	Model 3	Model 4
Control variables	Gender	0.012	–0.006	–0.006	–0.011
	Age	0.042	0.016	0.016	0.006
	Occupation	–0.025	–0.037	–0.038	–0.022
	Monthly income	0.021	0.011	0.011	–0.022
Independent variables	Performance expectancy		0.397 ***	0.397 ***	0.427 ***
	Price value		0.148 ***	0.148 ***	0.161 ***
	Privacy risk		–0.295 ***	–0.295 ***	–0.292 ***
	Social influence		0.436 ***	0.436 ***	0.431 ***
	Perceived threat		0.448 ***	0.448***	0.468 ***
Moderator variable	Medical technical knowledge			–0.002	–0.040
Interaction items	Performance expectancy * Medical technical knowledge				0.088 **
	Price value * Medical technical knowledge				0.148 ***
	Privacy risk * Medical technical knowledge				0.163 ***
	Social influence * Medical technical knowledge				0.084 **
	Perceived threat * Medical technical knowledge				0.263 ***
Statistics	R2	0.003	0.588	0.588	0.730
	Adjusted R2	–0.006	0.579	0.578	0.720
	F	0.333	64.196 ***	57.635 ***	72.021 ***

*** significant at the 0.001 level, ** significant at the 0.01 level, * significant at the 0.05 level.
